# 微芯片电泳技术在生物样品分离分析中的研究进展

**DOI:** 10.3724/SP.J.1123.2022.12004

**Published:** 2023-08-08

**Authors:** Jianying HUANG, Ling XIA, Xiaohua XIAO, Gongke LI

**Affiliations:** 中山大学化学学院,广东 广州 510006; School of Chemistry, Sun Yat-sen University, Guangzhou 510006, China

**Keywords:** 微芯片电泳, 生物样品, 分离分析, 研究进展, microchip electrophoresis, biological samples, separation and analysis, research progress

## Abstract

微芯片电泳技术是在微芯片中通过流体操控实现电泳分离的分析技术,具有分离效率高、样品量消耗少、分析速度快、易于集成等优势,已广泛应用于生物、医药等复杂样品的快速分离分析中。本文综述了微芯片电泳技术在微芯片材料、电泳模式、检测方式及生物样品分离分析等方面的研究进展。微芯片材料包括芯片材料与通道修饰方法以及电极材料与电极集成方式。芯片材料主要包括硅、玻璃、纸材料、聚二甲基硅氧烷和聚甲基丙烯酸甲酯等聚合物材料;通道修饰方法是指在微通道上的动、静修饰方法。电极材料与电极集成方式包括研制电极所需的金、铂、银材料以及将电极与微芯片集成的加工方式。根据施加电场是否均匀,微芯片电泳技术可分为均匀和非均匀电场两种电泳模式。均匀电场模式主要为微自由流电泳和微区带电泳,包括微等电聚焦电泳、微等速电泳、微密度梯度电泳等;非均匀电场模式主要为微介电泳。微芯片电泳技术通常与光谱、电化学和质谱等分析检测技术联用,实现复杂样品的快速、高效分离分析。近年来微芯片电泳在高通量和原位分离分析方面还发展了许多新模式和新策略。本文介绍了微芯片电泳技术在生物大分子、生物小分子、生物粒子等生物样品中的分离分析进展,并展望了微芯片电泳技术在生物样品分离分析中的发展趋势。

## Abstract (en)

Microchip electrophoresis is a separation technology that involves fluid manipulation in a microchip; the advantages of this technique include high separation efficiency, low sample consumption, and fast and easy multistep integration. Microchip electrophoresis has been widely used to rapidly separate and analyze complex samples in biology and medicine. In this paper, we review the research progress on microchip electrophoresis, explore the fabrication and separation modes of microchip materials, and discuss their applications in the detection and analysis of biological samples. Research on microchip materials can be mainly categorized into chip materials, channel modifications, electrode materials, and electrode integration methods. Microchip materials research involves the development of silicon, glass, polydimethylsiloxane and polymethyl methacrylate-based, and paper electrophoretic materials. Microchannel modification research primarily focuses on the dynamic and static modification methods of microchannels. Although chip materials and fabrication technologies have improved over the years, problems such as high manufacturing costs, long processing time, and short service lives continue to persist. These problems hinder the industrialization of microchip electrophoresis. At present, few static methods for the surface modification of polymer channels are available, and most of them involve a combination of physical adsorption and polymers. Therefore, developing efficient surface modification methods for polymer channels remains a necessary undertaking. In addition, both dynamic and static modifications require the introduction of other chemicals, which may not be conducive to the expansion of subsequent experiments. The materials commonly used in the development of electrodes and processing methods for electrode-microchip integration include gold, platinum, and silver. Microchip electrophoresis can be divided into two modes according to the uniformity of the electric field: uniform and non-uniform. The uniform electric field electrophoresis mode mainly involves micro free-flow electrophoresis and micro zone electrophoresis, including micro isoelectric focusing electrophoresis, micro isovelocity electrophoresis, and micro density gradient electrophoresis. The non-uniform electric field electrophoresis mode involves micro dielectric electrophoresis. Microchip electrophoresis is typically used in conjunction with conventional laboratory methods, such as optical, electrochemical, and mass spectrometry, to achieve the rapid and efficient separation and analysis of complex samples. However, the labeling required for most widely used laser-induced fluorescence technologies often involves a cumbersome organic synthesis process, and not all samples can be labeled, which limits the application scenarios of laser-induced fluorescence. The applications of unlabeled microchip electrophoresis-chemiluminescence/dielectrophoresis are also limited, and simplification of the experimental process to achieve simple and rapid microchip electrophoresis remains challenging. Several new models and strategies for high throughput in situ detection based on these detection methods have been developed for microchip electrophoretic systems. However, high throughput analysis by microchip electrophoresis is often dependent on complex chip structures and relatively complicated detection methods; thus, simple high throughput analytical technologies must be further explored. This paper also reviews the progress on microchip electrophoresis for the separation and analysis of complex biological samples, such as biomacromolecules, biological small molecules, and bioparticles, and forecasts the development trend of microchip electrophoresis in the separation and analysis of biomolecules. Over 250 research papers on this field are published annually, and it is gradually becoming a research focus. Most previous research has focused on biomacromolecules, including proteins and nucleic acids; biological small molecules, including amino acids, metabolites, and ions; and bioparticles, including cells and pathogens. However, several problems remain unsolved in the field of microchip electrophoresis. Overall, microchip electrophoresis requires further study to increase its suitability for the separation and analysis of complex biological samples.

微芯片电泳(microchip electrophoresis, MCE)技术从传统电泳法演变而来,可在微芯片上完成常规电泳的样品进样、电泳分离、分析检测等步骤,具有样品消耗少、分析速度快、自动化程度高、装置小型化、集成化等优点^[1⇓-3]^,广泛应用在化学、生物、医学和环境等领域^[4⇓⇓-7]^。MCE主要由微芯片材料模块、电泳模块、检测模块等组成,其易与光谱、电化学、质谱等检测器耦合,实现高通量、高灵敏的分析检测^[8⇓-10]^。随着3D打印技术的发展,具有复杂三维通道结构的微芯片可通过微型3D打印技术加工成型^[11⇓-13]^。Castiaux等^[14]^采用3D打印技术在4 h内完成了具有复杂三维结构的微芯片泵的设计和加工,并实现了在微米尺度上流体的精密操控。除了压力进样,MCE也可采用电动进样方式,但电动进样和压力进样都需要外加高压电源设备。Wang等^[15]^提出了一种简便的电泳滴定系统,无需外加高压设备即可对牛奶中的蛋白质进行可视化分析。

本文从微芯片材料、电泳模式、检测方式等方面介绍MCE的研究进展,并综述MCE在生物样品分离分析中的应用。图1总结了近10年来MCE在生物样品分离分析方面的文献报道情况,该研究领域每年有超过250篇的研究论文发表,正逐渐成为新的研究热点。生物样品主要可分为生物大分子、生物小分子和生物粒子,其中生物大分子主要包括蛋白质和核酸,生物小分子主要包括氨基酸、代谢物和离子,生物粒子主要包括细胞和病原体。

**图1:**
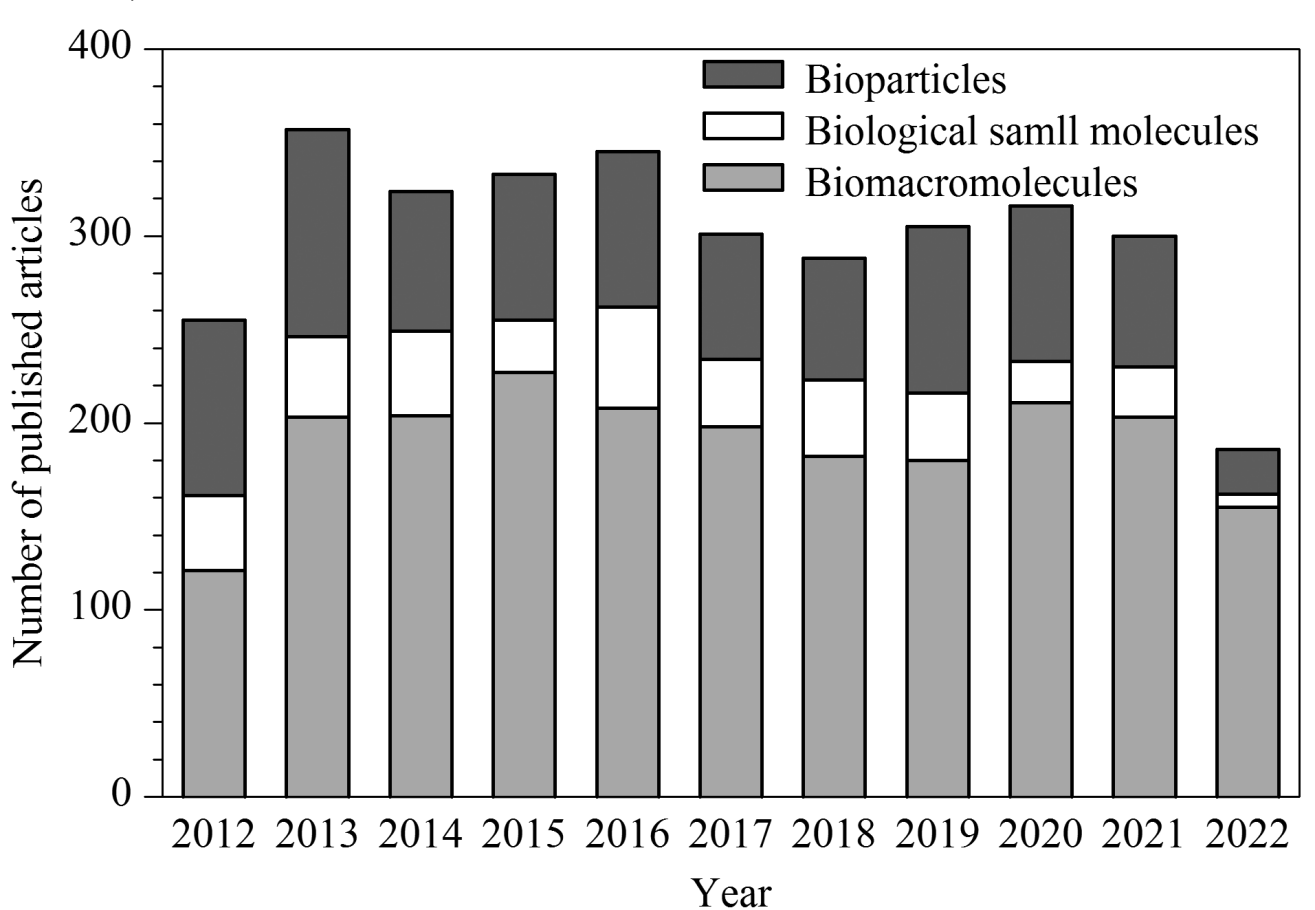
近10年MCE技术用于不同生物样品分离分析中的文献报道情况

## 1 微芯片电泳系统

MCE系统主要包括微芯片材料模块、电泳模块和检测模块,3个模块相辅相成,共同推动MCE向小型化、快速化两大目标发展。

### 1.1 微芯片电泳材料

#### 1.1.1 芯片材料与通道修饰方法

芯片材料需要满足可加工性、透明性、化学惰性、可修饰性等要求。目前芯片材料的研究主要围绕芯片材料选择及其加工工艺开展。不同材质芯片的优缺点和加工技术见表1。传统的电泳微芯片用硅和玻璃制造^[16]^,但硅易损坏,成本高,绝缘性和透光性较差,具有复杂的表面化学行为;而使用玻璃材质的制作工艺复杂,成本高且需要昂贵的仪器。近年来,微芯片的制造材料逐渐转向成本低、制备简单且对生物样品呈化学惰性的聚合物材料,如聚二甲基硅氧烷(polydimethylsiloxane, PDMS)、聚甲基丙烯酸甲酯(polymethyl methacrylate, PMMA)、环烯烃共聚物(copolymers of cycloolefin, COC)等^[17,18]^。聚合物材料还可以作为3D打印材料,其无需复杂的模具设备,使微流控设备的制造更加简单、快速^[19]^。Quero等^[20]^通过熔融沉积3D打印技术,采用聚对苯二甲酸乙二醇酯-1,4-环己烷二甲醇酯研制了通道尺寸为58 μm×65 μm(宽×高)的MCE芯片,成功分析了生物样品中K^+^、Na^+^、Li^+^的含量。立体光刻^[21]^、选择性激光烧蚀^[22]^、喷墨打印^[22]^等3D打印技术也成功用于微芯片的加工制备,实现了通道尺寸在200 μm以下的微芯片加工。纸也可以作为微芯片材料,一般通过立体光刻和喷墨打印进行通道加工^[23]^。与硅、玻璃等材质相比,纸芯片价格低,易于保存和运输,能够固定生物大分子,还可以通过简单的石墨沉积来制造电极,进一步降低MCE成本^[24]^。虽然芯片材料及其加工技术逐年完善,但仍存在制造成本高、加工时间长、使用寿命短的问题,阻碍了MCE的产业化发展。

**表1:** 不同材质芯片的优缺点和加工技术

Chip material	Advantages	Disadvantages	Processing technologies
Silicon	chemical corrosion resistance, good thermal stability, excellent strength and dissipation	fragile, poor insulation and light trans- mission, high cost	lithography, etching
Glass	easy surface modification, good electro permeability, light transmission and insulation, good biocompatibility	complex process conditions, high cost, long cycle, low bonding efficient	lithography, etching
Polymer	good chemical inertia, good optical properties, good thermal properties and electrical insulation, low cost	low surface hardness, poor heat resist- ance, hydrophobic surface	hot pressing, molding, 3D printing
Paper	low cost, good biocompatibility, self-drive ability, able to fix biomacromolecules	low surface strength, easy to evaporate, remaining and leaking in the chip channel	stereo lithography, inkjet printing, laser ablation

MCE通道中的电渗流(electroosmotic flow, EOF)会影响带电目标物的运动。石英、玻璃等材质的微芯片因硅羟基解离而产生带负电荷的Si-O^-^,吸引溶液中的正离子聚集在其周围,形成双电子层,在电解质水溶液和极性有机溶剂溶液中产生指向负极的EOF^[25]^,影响待测样品在通道中的运动。适当的表面修饰可以减少样品的吸附,调控EOF的大小和方向,从而提高MCE的分析能力。

MCE涂层可采用动态或静态修饰方式。动态修饰通常使用羟乙基纤维素等表面活性剂吸附在通道表面,与待测样品溶液形成动态平衡,这种修饰方式易于实现但稳定性较差;静态修饰则通过涂层材料的官能团与通道共价连接,或通过交联反应固定在通道表面,如玻璃微芯片通道通过表面硅烷醇基的硅烷化进行共价修饰,使其具有疏水性以减少吸附;聚合物可与聚乙二醇和聚丙烯酰胺通过静电或疏水相互作用进行非共价连接^[26]^,与动态修饰相比,静态修饰的结构更稳定但不可回收。Sun等^[27]^利用甲基丙烯酸二甲酯和聚甲基丙烯酸缩水甘油酯组成的共聚物完成了PDMS微芯片的超亲水性修饰,涂层修饰在1 h内即可完成,修饰后的PDMS可抑制样品吸收,且EOF的影响可忽略不计。Song等^[28]^将PDMS浸入不同含量(2.5%~10%)的聚乙二醇磷酸盐缓冲溶液中,测量并比较了聚乙二醇改性PDMS通道的Zeta电位值。结果表明,PDMS表面的Zeta电位与聚乙二醇的含量成反比,当聚乙二醇含量增加到5%以上时,Zeta电位具有平台效应。我们课题组^[29]^结合动态和静态修饰来准确控制通道中的EOF,在COC材料的MCE系统上实现了化妆品中肌肽和烟酰胺的快速、同时定量检测(图2)。目前聚合物通道表面的静态修饰手段较少,且大多数都是通过物理吸附与聚合物相结合,仍需开发高效的聚合物通道表面修饰方法。并且,无论是动态修饰还是静态修饰,都需要引入其他化学物质,可能不利于后续实验的开展。

**图2:**
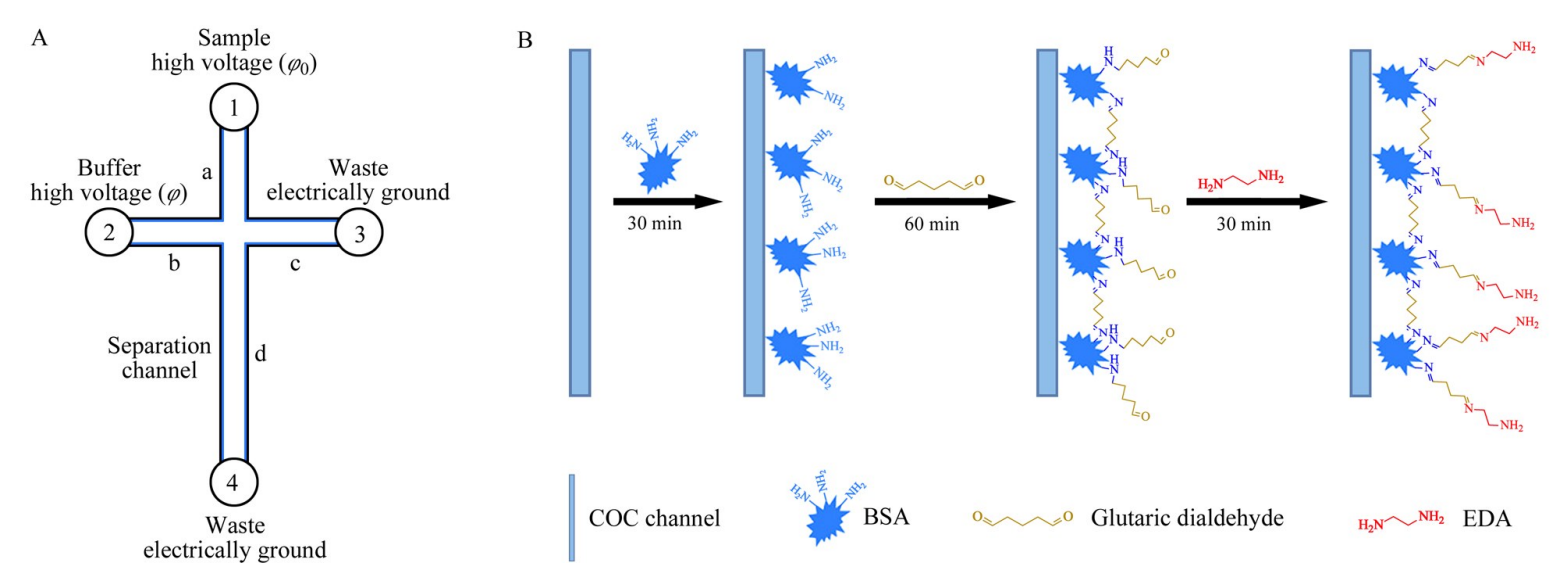
MCE芯片和COC材料表面修饰过程示意图^[29]^

#### 1.1.2 电极材料与电极集成方式

与常规电泳相比,将电极应用到微芯片等器件中需要考虑成本、电导率、材料反应性以及制造时间和复杂性等变量。金、铂和银是常见的电极材料^[30⇓-32]^,银的导电性强且成本低,但易被氧化而受到影响;将铂丝直接插入微芯片电极孔中来施加电压是最简单的电极集成方式,但会导致样品污染^[33]^。为了解决这个问题,并实现微芯片器件的大规模生产,可在加工过程中将电极集成到微芯片上,这些加工方法主要包括金属化、光刻、电极剥离法、丝网印刷法等^[34,35]^,尤其是丝网印刷法,已被证明是快速、经济、成本效益更高的电极集成方法^[36]^。Petroni等^[37]^仅通过胶带将导电碳材料手动沉积到丙烯酸材料上,所使用的碳墨水电极制造成本低,无需专用设备即可轻松复制,展示了丝网印刷的经济性。碳电极材料可以扩展到其他类型的墨水,以便针对其他应用进行特定使用。虽然电极与微芯片的集成降低了重复使用电极造成的样品污染风险,但其用于MCE上的生物样品分析通常是一次性的。贵金属电极的成本较高,通过外部电极与微芯片一次性导电膜结合可降低成本与样品污染风险。Liu等^[38]^制造了一种通过物理保护层隔绝电极和样品池的MCE装置,用分析样品与带有印刷碳电极的薄膜相连代替传统贵金属电极,实现电动传输。总的来说,目前电极与微芯片的集成加工工艺较为复杂,简单高效的集成工艺仍是目前的研究热点。

### 1.2 微芯片电泳模式

MCE基于带电分析物在电场中的运动进行分离分析,电场大小及是否均匀是影响样品分离分析的关键因素,其中前者是实验中控制电渗速度的关键参数,后者决定了MCE模式。MCE电泳模式可分为均匀电场模式和非均匀电场模式,其中均匀电场模式主要为微自由流电泳和微区带电泳;非均匀电场模式主要为微介电泳。MCE电泳模式的原理见图3。

**图3:**
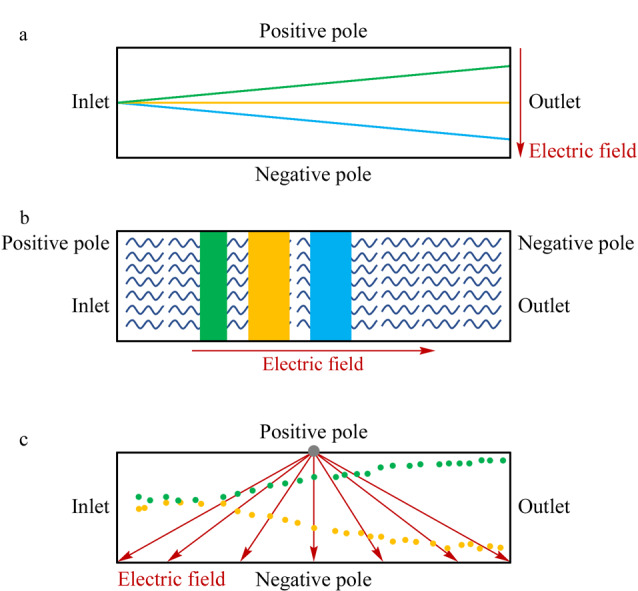
MCE电泳模式原理示意图

#### 1.2.1 均匀电场

微自由流电泳和微区带电泳是基于均匀电场分离带电样品的两种经典电泳模式。微自由流电泳可分为微等电聚焦电泳、微等速电泳、微密度梯度电泳等。微自由流电泳可连续分离复杂生物样品,但通常需要较长的缓冲液清洗和优化过程,前处理过程非常耗时。Shebindu等^[39]^提出了一个可编程微流控平台,可实现多种生物样品全自动优化的微等速电泳,他们采用可升降微阀技术,设计并制造了由二维微阀阵列组成的微流控平台,与MCE芯片无缝集成;其中微阀阵列平台用于基本液体操作,如计量、混合、选择、输送和清洗程序,简化了生物样品的缓冲液选择和验证过程。微区带电泳包括微凝胶电泳、微薄层电泳、微滤纸电泳等,适用于荷质比相近但相对分子质量不同的生物大分子的分离分析。传统的聚丙烯酰胺凝胶等材料虽然可保留蛋白质的折叠状态,但其分辨率较差。热凝胶电泳采用黏度随温度变化的热响应聚合物,如Pluronic F-127凝胶等,不仅可以简化实验过程,还可以实现高分辨率。如Cornejo等^[40]^使用热凝胶电泳定量分析多个miRNA时,通过在序列上设计与4个目标miRNA互补的荧光探针和具有锥形几何形状的微通道,将分析物和探针直接混合在热凝胶中并加载到微通道内,在通道上施加电压即可实现miRNA的在线预富集和分离,无需复杂的前处理过程。该方法能够在5 min内实现多个miRNA的分析,检出限(limit of detection, LOD)低至10^-11^ mol/L。微等速电泳也可与热凝胶电泳联用,使样品浓缩与分离同时进行^[41]^,两种电泳模式联用也可实现蛋白质对不同堆叠和分解区域的要求,具有更高的分离效率和更短的分析时间,适用于天然蛋白质变体的分析。

#### 1.2.2 非均匀电场

微介电泳(dielectrophoresis, DEP)是通过非均匀电场中产生极化力将悬浮颗粒与悬浮液分离的典型方法^[42]^。与均匀电场下的电泳不同,DEP中的样品可以不带电荷,但必须在电场中可极化并形成电偶极子,通过作用在偶极子上的DEP力推动样品向电场较强或较弱的一方移动。DEP具有无需标记、快速和准确的独特优势,广泛应用于生物分子诊断、医学和聚合物研究的微流控领域^[43⇓-45]^。MCE中DEP力的形成与控制主要依赖于电极形状和微通道结构。Muhsin等^[46]^通过两组平行连接的聚焦电极和三组叉指电极阵列组成的复合电极来产生DEP力,迫使细菌向微通道中心线移动,当细菌病原体到达检测区域时,其浓度较高,检出限为3个细菌细胞/mL。Zhang等^[47]^设计了一种带有Y-Y型结构的MCE系统,用于DEP分离肺癌细胞。微通道上由连续的三角形组成,当细胞通过通道时,受到不同强度的DEP力作用,诱导细胞迁移,达到静电平衡,从而实现循环肿瘤细胞和红细胞的分离,分离效率可达99%。DEP技术在临床和生物医学等流体筛分中得到应用^[48]^,可实现对同一物种微生物的超快速、高效和选择性捕获和分离。

### 1.3 微芯片电泳检测方式

由于芯片通道尺寸通常是微米级、样品量通常是纳升到微升级,微芯片电泳对检测器的性能如灵敏度、响应速度等要求较高。微芯片电泳常用的检测方式包括光学检测、电化学检测、质谱检测和激光诱导荧光(laser induced fluorescence, LIF)检测,其中,LIF检测具有背景噪声低、灵敏度高和易于在芯片上实现的特点,与MCE结合可广泛应用于神经递质、抗生素、多肽、细胞检测等众多领域^[49,50]^。在LIF检测中,微芯片通道与检测器的对准过程对于荧光信号最大化和检测灵敏度至关重要,但LIF所需的荧光标记往往需要繁琐的有机合成过程,且并不是所有的样品都能够被标记,限制了LIF的使用场景。Scott等^[51]^开发了一种用于MCE通道自动校准的方法,该方法不需要荧光染料和额外硬件,仅通过激发期间从通道发射的固有光学特征即可在自由旋转的微流体设备上定位宽度为5~50 μm的分离通道。智能算法的引入在很大程度上弥补了MCE从集成化到智能化之间的差距,推动了生物样品分析过程的全自动化,与不同检测方式的耦合使MCE易实现高通量检测和原位检测^[52,53]^;前者解决了微流控技术的低通量问题,后者则体现了MCE的便携性,为高通量、便携式生物样品检测系统的构建提供了思路。无需标记的MCE-化学发光法和微介电泳法适用范围较为局限,如何简化实验流程,实现简单、快速的MCE分析仍是一个难题。

#### 1.3.1 高通量检测

尺寸的小型化使MCE在微量和快速分离分析中具备优势,但相应的也容易受到样品分析通量的限制,尤其是在多核酸检测等方面。早期通常采取提高微通道数量的方法,如4通道、8通道甚至16通道并行来弥补传统MCE通量上的不足,但会使微通道结构及微芯片材料选择和加工工艺更加复杂,且多通道并行也会给检测器的耦合带来问题。近年来,一些新颖的高通量MCE系统相继得以报道。Pendharkar等^[54]^采用基板剥离法研制了带有6000个微型阵列孔的PDMS微芯片,该芯片在每次实验中可操作约6000个单细胞或细胞对,将该芯片用于CT26和骨髓树突状细胞之间的电融合,成对细胞之间的融合效率高达70%(3000~3500个细胞)。Grist等^[55]^采用MCE结合三维单细胞免疫印迹技术,在光活性聚丙烯酰胺凝胶表面设计了微孔阵列,用于细胞分离和裂解。三维结构的引入可提高微孔阵列的密度,从而提高检测通量。每个微孔中的单细胞裂解物通过电泳分开投射到三维凝胶中,并在凝胶中捕获照片,用于免疫探针和共焦光片成像。该系统同步了数百个哺乳动物乳腺和脑肿瘤细胞的细胞质和核蛋白分析,电泳通量>2.5个细胞/s,比传统的连续取样法快70倍。新的分析策略引入可减少实验步骤并增加检测通量。Quan等^[56]^研制了由反转漏斗通道结构、纯水凝胶电泳分离介质集成和计算机辅助流体模拟组成的MCE系统,发展了高分辨率的多维全蛋白天然微芯片电泳技术。以大肠杆菌、金黄色葡萄球菌和枯草芽孢杆菌为模型分析物,利用新的图像分析算法可对纯样品或混合样品进行微生物定量和半定量分析,并通过扇形或圆形通道布局来增加通道阵列的密度,进一步提高峰值容量,在1 min内实现了高分辨率全蛋白MCE分析。MCE的高通量分析往往离不开复杂的芯片结构和较为繁琐的检测方式,简单的高通量分析技术还有待研究。

#### 1.3.2 原位检测

MCE系统的模块化提升了其便携性,使之更适用于生物样品的原位检测。Mayor等^[57]^受乐高玩具的启发,设计了一种新型的MCE结合LIF检测系统。该系统由用户自定义的模块构建,以类似于乐高积木的插拔式方法快捷连接光学和电泳等微流控部件,其成本比商业购买价格便宜70%以上,并应用于糖蛋白衍生聚糖的分析,具有很好的原位检测普适性。Guo等^[58]^报道了一种具有多种电场调节功能的可视便携式MCE系统,该系统可以采用模块化设计实现各种基本微流体实验的电场调节功能,也可通过更换模块组实现交替电渗、介电电泳等功能。在电渗粒子聚焦实验中,它通过智能手机显示微流控芯片的内部反应过程,使用手机应用程序分析实验结果,得到了与数值模拟一致的结果。Lin等^[59]^报道了一种基于交流电电热(AC electrothermal, ACET)效应来设计人体上生物流体流动轮廓的可穿戴电子流体驱动装置,通过电脑编程精确驱动微芯片中的微流体,并实现了基于芯片的实验室样品制备和人体操作,非常适用于人体生物样品的原位在线分析,为实现MCE的穿戴式复杂分析创造了新的可能性。

## 2 微芯片电泳在生物样品分离分析中的应用

MCE的样品消耗量少,分析时间快,分离效率高,且样品制备和检测集成在微芯片上,适用于快速分析和现场检测,近年来在生物、食品、环境、临床医学、药学等领域得到了广泛应用(表2)。以下介绍MCE在生物大分子、生物小分子、生物粒子等生物样品中的分离分析进展。

**表2:** MCE在生物样品分离分析中的应用

Samples	Analytes	Material	Mode	Detection method	LOD	Ref.
Cell lysate	miRNA-141	glass	uniform	LIF	0.8×10^-15^ mol/L	[60]
Model mixture	fructose	glass	uniform	CD	1.5×10^-4^ mol/L	[61]
	galactose				1.8×10^-4^ mol/L	
	glucose				3.0×10^-4^ mol/L	
	lactose				2.2×10^-4^ mol/L	
	sucrose				7.4×10^-4^ mol/L	
Antiepileptic drug	vigabatrin, pregabalin,	glass	uniform	LIF	0.6×10^-12^ mol/L	[62]
	gabapentin					
Human urine	dopamine	glass	uniform	LIF	1.7×10^-9^ mol/L	[63]
	norepinephrine				2.4×10^-9^ mol/L	
	serotonin				2.7×10^-9^ mol/L	
Single-cell lysate	animal proteome	glass	uniform	CD	/	[64]
Cell lines	mitochondria	PDMS	non-uniform	CD	/	[65]
Blood serum sample	preterm birth biomarkers	COC	uniform	LIF	1.2×10^-9^ mol/L	[66]
Human urine	sialylated, *N*-glycan	COC	uniform	MS	/	[67]
Foodstuffs	carminic acid	PMMA	uniform	FL	6.9×10^-10^ mol/L	[68]
Human urine	*α*-fetoprotein	PDMS	uniform	FL	7.2×10^-9^ g/mL	[69]
Drinking water, milk	*E. coli*	PDMS	uniform	LIF	3.7×10^2^ CFU/mL	[70]
Glycan, glycopeptide	glycoprotein	PMMA	uniform	MS	/	[71]
Food	*E. coli*	PC	non-uniform	AD	1.0×10^2^ CFU/mL	[72]
	*S. enteritidis*				1.0 CFU/mL	

*E. coli*: *escherichia coli* O157∶H7; *S. enteritidis*: *Salmonella enteritidis*; PDMS: polydimethylsiloxane; PMMA: polymethyl methacrylate; PC: polycarbonate; LIF: laser induced fluorescence; CD: conductivity detector; FL: fluorescence; AD: amperometric detection; /: not detected; CFU: colony forming units.

### 2.1 生物大分子

生物大分子样品量少且对其进行分离分析一般需要较多步骤,如DNA的萃取和纯化、聚合物链式反应(polymerase chain reaction, PCR)扩增、电泳分离和检测等,MCE可以在一块芯片上完成几乎所有步骤,非常适用于核酸等大分子的分离分析^[73]^。20世纪90年代,Woolley等^[74]^就采用MCE技术实现了高速、高通量的DNA测序。之后,Anazawa等^[75]^研制了基于单边入射激光锯齿形多通道DNA的高灵敏MCE芯片,通过微自由流电泳结合多个LIF检测器同时检测8个通道中的DNA链片段,在10 min内单碱基分辨率达到300,实现了17个位点的短串联重复序列基因分型。如图4所示,Yang等^[76]^以核酸适配体为目标探针,将其与目标分子特异性结合,从而触发级联信号放大反应,并结合核酸外切酶信号扩增技术生成具有荧光标记的目标DNA片段,建立新的MCE-化学发光检测方法,实现了人体血浆样本中*γ*-干扰素的超灵敏检测,检出限较传统MCE-化学发光法低7个数量级。MCE在RNA的快速、高灵敏检测中也有较多研究。Wei等^[60]^采用分离辅助双循环信号放大策略开发了一种基于微凝胶电泳的细胞裂解液中微量miRNA的超灵敏MCE-LIF检测方法,当以人膀胱癌细胞裂解液中提取的miRNA-141为模型分析物时,检出限可低至8.0×10^-15^mol/L。将MCE与探针延长扩增技术巧妙结合所设计的特异性探针^[77]^,可显著提高5种细菌中16S rRNA基因的检测灵敏度,检出限低至3.0×10^-14^mol/L。在蛋白质分析检测方面,Hennig等^[78]^开发了基于微滤纸电泳的MCE系统,将电泳的微观结构印刷到醋酸纤维素薄膜上进行连续免疫学分析,在15 min内分离了分子量为15~120 kDa的荧光标记蛋白,样品消耗量减少了100倍。Ouimet等^[79]^利用目标物与缓冲液间的密度差异实现了MCE装置的自动进样,成功用于蛋白质-蛋白质相互作用样品和酶样品的分离。该装置平台搭建简单,可以连续使用1000次,每次样品在10 s内可实现分离。此外,还可以利用MCE技术建立高通量过滤板实验,测定牛血清蛋白、白喉毒素突变体溶菌酶的多组分吸附等温线,并研究它们与疏水树脂的相互作用^[80]^。

**图4:**
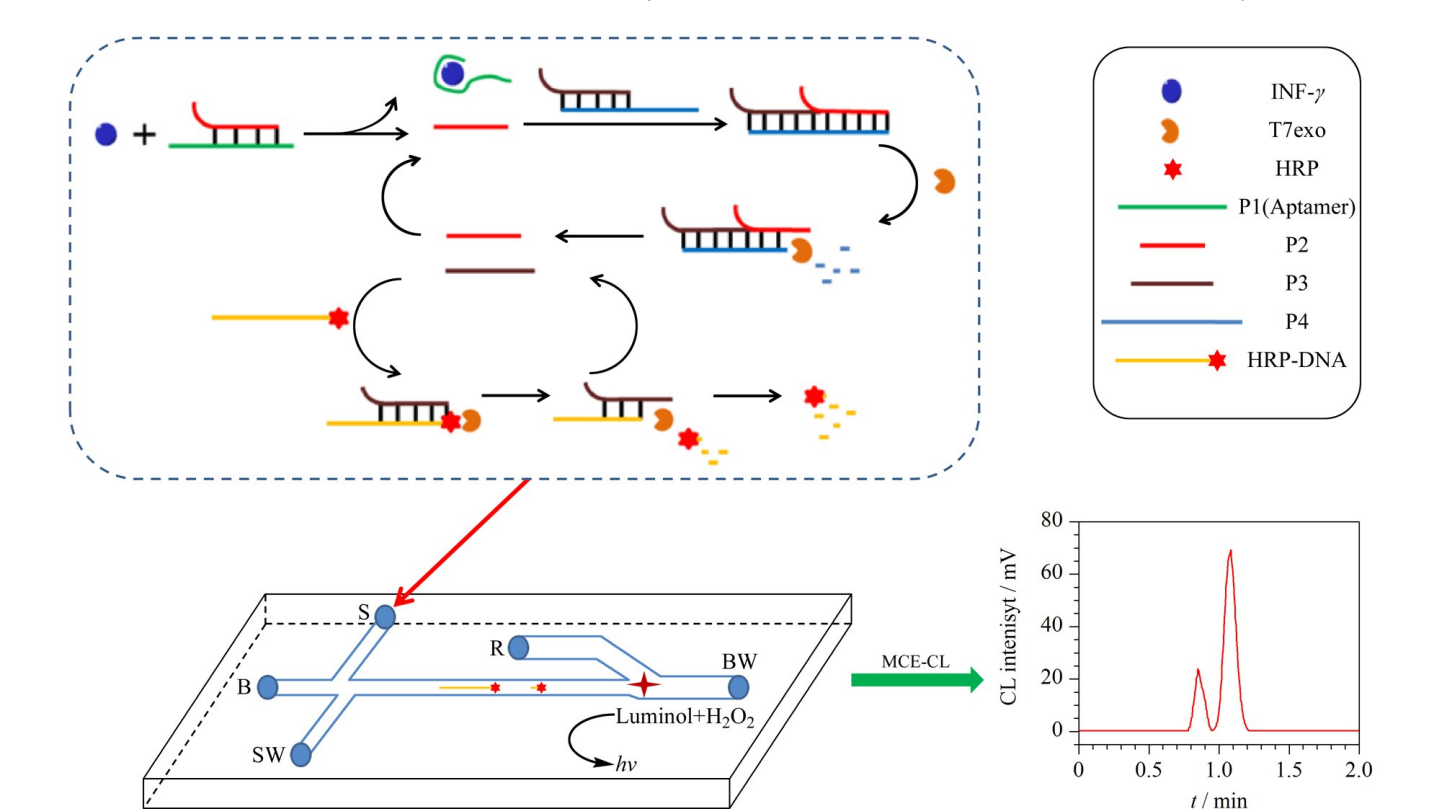
基于级联信号放大的MCE-化学发光法示意图^[76]^

### 2.2 生物小分子

不同分离模式的MCE可用于大多数小分子化合物,如药物残留、代谢物、氨基酸等的分离分析,尤其是低浓度、少量的生物样品分析。Masár等^[68]^建立了一种用于测定多种药物配方中非甾体类消炎药含量的MCE方法,他们在pH 6.5的芯片第一分离通道进行等速电泳,在pH 7.0的芯片第二分离通道进行区带电泳,通过在线结合等速电泳和区带电泳,实现目标物的富集和杂质的去除,成功用于11种商业药剂中3种常见非甾体类消炎药含量的分析。Cabot等^[81]^采用3D打印技术研制了用于直接分析尿液中低丰度核黄素代谢物的模块化MCE装置,核黄素代谢物可在2 min内通过微区带电泳完成分析。将MCE-质谱法用于动态监测10天内仓鼠卵巢细胞培养基中的氨基酸浓度变化,在2 min内可实现16种氨基酸的快速分离与高灵敏检测^[82]^。将基于微区带电泳的MCE和化学发光法结合后,由于谷胱甘肽上的巯基对鲁米诺-H_2_O_2_体系有强敏化作用,无需通过重氮鲁米诺进行细胞内标记即可实现大鼠肝脏单个细胞中谷胱甘肽的定量检测,单个细胞的分析可在2 min内完成,LOD为9.6×10^-7^ mol/L^[83]^。

### 2.3 生物粒子

MCE可以在微尺度上精确、重复地操纵细胞和病原体等生物粒子,为生物粒子分析提供了重要的技术平台^[84,85]^,尤其是DEP可在外加非均匀电场下控制细胞的运动来操作细胞和病原体,从而被广泛应用。如Park等^[72]^将基因扩增、溶液混合和电化学检测集成到微芯片中,应用于大肠杆菌、肠炎链球菌等病原体的检测,具有较高的重复性(约10%的误差)和灵敏度,LOD分别为5.0×10^-11^ g/mL和5.0×10^-12^ g/mL,降低了病原体评估的成本和检测时间。将PCR和MCE结合可用于伤寒沙门氏菌、金黄色葡萄球菌、大肠杆菌3种病原体的同时检测。采用3对引物对从3种病原菌中提取的目标基因进行PCR扩增,标记的PCR产物在135 s内即可电泳分离,3种细菌的LOD均小于62 CFU/mL^[86]^。进一步将多重PCR与MCE-LIF结合用于检测牛奶样品中的大肠杆菌、鼠伤寒沙门菌和单核细胞增生杆菌,实验所需的分析时间更少,得到的检出限更低^[87]^。Li等^[88]^使用两个不混溶相(聚乙二醇和葡聚糖)在微通道中形成液-液界面,通过目标颗粒和细胞在液-液界面上的微介电泳实现了粒子和细胞的连续分离,为粒子和细胞的分选提供了新思路。

## 3 结论

MCE具有样品消耗少、分析时间快、分离效率高且易于集成整合的优点,非常适用于生物样品的快速、高灵敏、现场检测。但MCE的芯片制作条件比较苛刻,芯片通道长度有限,实验重复性差,有待改进;生物分子非常容易吸附在微芯片通道表面,复杂样品基质对MCE的分离性能影响较大。因此,MCE技术在生物等复杂样品分离分析中还有诸多关键问题有待深入研究。未来,MCE可在以下几个方面实现更好的发展:(1)改善微芯片的加工方法,在保证工艺流程的便捷性下实现微芯片的高成品率;(2)开发用于多重分析的高通量微芯片装置;(3)开发更为简便的涂层修饰方法,抑制目标物在芯片通道上的吸附,改善电渗;(4)将高效分离富集技术、微流控技术与智能手机等便携式装置相结合,实现复杂样品的在线、现场、高效分析检测。
